# Correction to: Automated classification of cytogenetic abnormalities in hematolymphoid neoplasms

**DOI:** 10.1093/bioinformatics/btac826

**Published:** 2022-12-29

This is a correction to: Andrew Cox, Chanhee Park, Prasad Koduru, Kathleen Wilson, Olga Weinberg, Weina Chen, Rolando García, Daehwan Kim, Automated classification of cytogenetic abnormalities in hematolymphoid neoplasms, *Bioinformatics*, Volume 38, Issue 5, 1 March 2022, Pages 1420–1426, https://doi.org/10.1093/bioinformatics/btab822

In the originally published version of this manuscript, the funder was incorrectly identified as the Cancer Prevention Research Institute of Health. This should be the Cancer Prevention Research Institute of Texas.

This error has been corrected online.

